# Building CNN-Based Models for Image Aesthetic Score Prediction Using an Ensemble

**DOI:** 10.3390/jimaging9020030

**Published:** 2023-01-29

**Authors:** Ying Dai

**Affiliations:** Faculty of Software and Information Science, Iwate Prefectural University, Takizawa 020-0693, Japan; dai@iwate-pu.ac.jp

**Keywords:** aesthetic score prediction, CNN architecture, ensemble, photography composition principle, attention region

## Abstract

In this paper, we propose a framework that constructs two types of image aesthetic assessment (IAA) models with different CNN architectures and improves the performance of image aesthetic score (AS) prediction by the ensemble. Moreover, the attention regions of the models to the images are extracted to analyze the consistency with the subjects in the images. The experimental results verify that the proposed method is effective for improving the AS prediction. The average F1 of the ensemble improves 5.4% over the model of type A, and 33.1% over the model of type B. Moreover, it is found that the AS classification models trained on the XiheAA dataset seem to learn the latent photography principles, although it cannot be said that they learn the aesthetic sense.

## 1. Introduction

Automatic image aesthetic assessment (IAA) can be applied to a variety of tasks, such as image recommendation, image retrieval, photo management, photography training and product design (cooking). Deng, Loy, and Tang (2017) [1] present an experimental survey about this field’s research. In this paper, besides a discussion of the state-of-the-art research, the authors show that deep learning is a powerful tool for aesthetic scoring. Early efforts in IAA focus on extracting designed hand-crafted features, according to the known photographic principles, for example, the rule of thirds, color harmony, and global image layout [2,3,4,5]. With the advance of the convolutional neural network (CNN), recent methods have aimed to map image aesthetics to different types of tasks using CNNs, particularly including high/low quality classification, aesthetic score prediction and their distribution [6,7,8,9,10,11].

Recently, in order to improve the accuracy of IAA, Sheng, Dong, Ma, Mei, Huang and Hu (2018) [12] proposed a multi-patch aggregation method for image aesthetic assessment, while preserving the original aspect ratio. Zhang, Gao, Lu and He (2019) [13] proposed a gated peripheral–foveal convolutional neural network with a double-subnet architecture. In Ref. [14], the same authors propose a novel multimodal recurrent attention CNN, which incorporates the visual information into the text information. In Refs. [15,16], the contributions of different regions at the object level to aesthetics are adaptively predicted. However, the above methods have not seemed to improve the results particularly well.

She, Lai, Yi and Xu (2021) [17] propose a method of utilizing a hierarchical layout-aware graph convolutional network to capture layout information for unified IAA. However, although there is a strong correlation between image layouts and perceived image quality, the image layout is neither the sufficient condition, nor the necessary condition, to determine an image’s aesthetic quality. In fact, several typical failure cases presented in Ref. [17] confirm the above statement. Some pictures appear to have good layouts that seem to meet the rule-of-thirds and are predicted to have a high rating. However, the ground truths (GT) of these images have a low rating. One picture seems not to meet the photography composition principles and is assigned a low rating; however, its GT are of a high rating.

Generally, modeling IAA is performed by supervised learning. To train the model, most of the research utilizes the labeling data of images regarding aesthetics in a public photo dataset, such as CUHK-PQ [1] or AVA [18]. However, these aesthetic data are generally labeled by amateurs. Whether the labeling data embody the latent principles of aesthetics is not clear. Therefore, whether the IAA models trained on these datasets are significant is also unclear. To make the labelled data embody the photo’s aesthetic principles, Dai (2020) [19] aims to establish a photo dataset called XiheAA, in which photos are scored by an experienced photographer; this is because it is assumed that the experienced photographers should be more able to reflect the latent principles of aesthetics when they assess the photos. These labelled images are used to train the IAA model. However, the IAA exhibits a highly skewed score distribution. In order to solve the imbalance issue in aesthetic assessment, in this paper, the author proposes a method of repetitive self-revised learning (RSRL) to retrain the CNN-based aesthetic score prediction model repetitively by transfer learning; this is to improve the performance of imbalance classification, caused by the overconcentrated distribution of the scores. Moreover, Dai (2022) [20] focuses on the issue of CNN-based RSRL to explore suitable metrics for establishing an optimal model of IAA. Further, the first fixation perspective (FFP) and the assessment interest region (AIR) are defined by the feature maps of the IAA model, so as to analyze whether the feature maps reveal the photography principles. Although several experiments have shown effectiveness of the RSRL on the imbalance classification, the ways in which to construct an aesthetic score prediction model that really embodies the aesthetic principles on the IAA is not involved.

In photography, it is known that the two important elements of assessing a photograph are the subject and the holistic composition. One standard for a good photograph is that the image should achieve attention-subject consistency. Inspired by the above knowledge, we propose a framework that constructs two types of IAA models with different CNN architectures, and improves the performance of image AS prediction by the ensemble, so as to solve the limitations of the state-of-the-art research mentioned above. Moreover, the consistency of the subject in the photo with the attention regions of the models is analyzed. The contributions of this paper are summarized as follows.

- Besides fine-tuning the pretrained models, a new CNN architecture that could embody the holistic composition of the image is designed. Based on this architecture, the models with different architectural parameters are trained on the XiheAA dataset [19], in order to predict an image’s aesthetic score.
- The performances of the above models are evaluated, and an ensemble method of aggregating two models is proposed to improve the performance of the AS prediction.
- The feature maps of the models regarding the images are analyzed. It is found that the attention regions of the models are often consistent with the subjects of the images, and follow the simple photography composition guidelines, such as visual balance, and the rule of thirds, if they are predicted to have the high aesthetic scores, otherwise the opposite, whether the predictions are correct or not. It is indicated that the models trained on XiheAA seem to learn the latent photography composition principles, but it cannot be said that they learn the aesthetic sense.

## 2. Related Works

Image Aesthetics Assessment (IAA) Besides the research mentioned in the Section Introduction, the other main-stream research on IAA is the following. 

Lee and Kim (2019) [14] propose a unified algorithm to solve the three problems of image aesthetic assessment: score regression, binary classification, and personalized aesthetics. Moreover, the personalized regression model is also trained on the FLICKERAES dataset [21]. However, the mean of the five workers’ scores was used as the ground truth score. Accordingly, it is not clear that the predicted score embodies the inherently personal aesthetics.

On the other hand, some researchers aim to extract and analyze the aesthetic features to find the relation with the aesthetic assessment. Jang and Lee (2021) [22] present an in-depth analysis of the deep models. The authors find that the extracted features for aesthetic classification are largely different from those for image classification. In Ref. [23], besides extracting deep CNN features, Li, Li, Zhang and Zhang (2020) propose five algorithms for extracting handcrafted aesthetic feature maps. The aesthetic features and CNN features are fused to improve the aesthetic assessment by designing a novel feature fusion layer. However, the experimental result shows that the fusion only improves the accuracy by 1.5%, compared to no fusion. Accordingly, it is necessary to investigate whether the incorporation of the inefficiently hand-crafted aesthetic features with the deep CNN features is needed. 

Recently, the fusion technologies have been focused on improving the accuracy of the aesthetics assessment. Varga (2022) [24] introduces a novel, deep learning-based architecture that relies on the decision fusion of multiple-image quality scores, coming from different types of convolutional neural networks. Takimoto, Omori and Kanagawa (2021) [25] propose an aesthetic assessment method that is based on multi-stream and multi-task convolutional neural networks. Varga (2022) [26] defines an optimization problem using the weighted sum of a few IAA metrics. The experimental results show that the proposed methods can effectively estimate perceptual image quality on four large IAA benchmark databases, such as LIVE [27], TID2013 [28], TID2008 [29], and CSIQ [30]. 

On the other hand, applying an image aesthetic assessment to the design field has become a hot topic in recent years [31,32,33,34]. For example, Jin, Chen and Zhou (2021) [31] analyze the impact of cover image aesthetics on the willingness to read the content. Sheng, Dong, Huang, Chai, Zhang, Ma and Hu (2021) [32] propose a method for food image assessment by learning its visual aesthetics. Khajehabdollahi, Martius and Levina (2019) [33] propose a method for generating abstract images by using a correction structure with the aesthetics.

**Figure 1:**
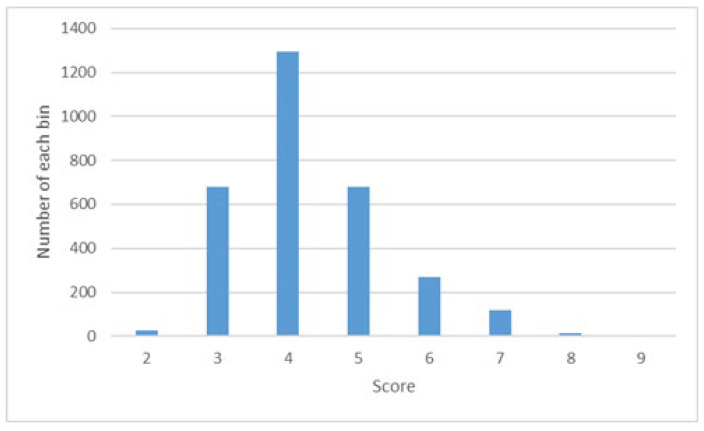
Score distribution of the XiheAA dataset.

XiheAA dataset [19] This dataset contains 3100 photos, aesthetically scored by an experienced photographer. The photos were taken by the students of the photographer’s on-line class. The type of equipment used to take the photographs was various, including mobile cameras, digital cameras, and SLR cameras. The average time of experience in the photographer’s class was approximately one year. The scores range from two to nine. Therefore, the number of classes *N* = 8. The distribution of the scores is shown in Figure 1.

RSRL [19] The approach of the CNN-based RSRL is to drop out the low likelihood samples of the majority classes of scores repetitively; this is in order to overcome the inference of these samples in the minority classes and prevent the loss of the samples with discriminative features in the majority classes. In this process, the models are re-trained by transfer learning iteratively, until the F-measure reaches the maximum.

FFP and AIR [20] According to the photography principles, people usually focus on the most salient object and its relations with the other elements when enjoying the pictures. The most salient object is believed to be the first fixation perspective (FFP), while the relation region that influences the aesthetic assessment is considered to be the assessment interest region (AIR). For a CNN-based IAA model, it is supposed that the most activated feature map should be related to the FFP of the image, and the sum of the feature maps should be related to the AIR.

Ensemble deep learning Ganaie, Hu, Malik, Tanveer and Suganthan (2022) [35] review the state-of-the-art deep ensemble models. The ensemble models are broadly categorized into bagging, boosting, stacking, negative correlation-based deep ensemble models, explicit/implicit ensembles, homogeneous/heterogeneous ensemble, and decision fusion strategies-based deep ensemble models. The applications of deep ensemble models in different domains are also briefly discussed. In Thurnhofer-Hemsi, Lopez-Rubio, Dominguez, Elizondo (2021) [36], an ensemble of improved convolutional neural networks is combined with a test-time regularly spaced shifting technique for skin lesion classification.

## 3. Methodology

### 3.1. Overview

**Figure 2:**
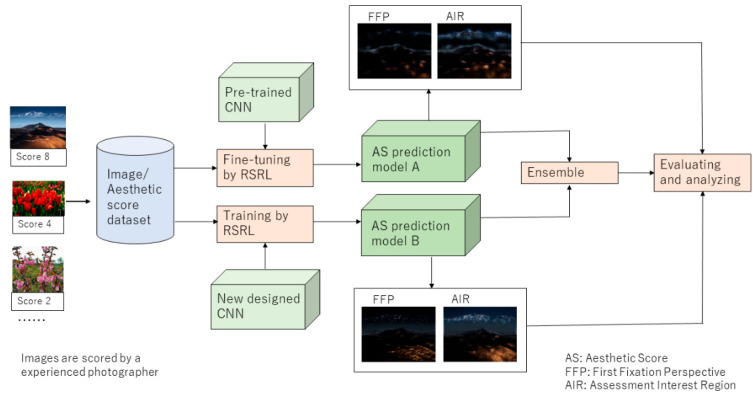
An overview of the proposed method.

According to the photography principles, an overview of our proposed framework, which constructs two types of IAA models with different CNN architectures and improves the performance of the image AS prediction by the ensemble, is shown in Figure 2.

For this framework, two types of CNN-based models are trained by RSRL on XiheAA dataset. Model A is expected to extract the subject of the image for predicting the image’s aesthetic score (AS), and model B is expected to extract the holistic composition for the prediction. Because the pretrained models, including alexNet, resNet18, and efficientNetB0, are trained on ImageNet following the general classification task, and, therefore, cover a wide range of content, it is suitable to use such models to construct model A by transfer learning on the XiheAA dataset Dai [19], meanwhile, the number of classes is changed from 1000 to 8, because the scores rated on the XiheAA dataset are in the range of [2,9]. On the other hand, because the XiheAA dataset is rated by an experienced photographer, it is considered that a new designed CNN that is trained on it could construct model B, which reflects the holistic composition of the images. Moreover, on the basis of the photography principles, the ensemble of model A and model B is applied to improve the performance of the prediction. Next, the FFPs and AIRs of model A and model B are computed to analyze the consistency of the attention regions of the models with the photography composition principles. In the following section, the architecture of the new designed CNN, the method of the ensemble, and the FFPs and AIRs of the images, regarding model A and model B, are explained in detail.

### 3.2. New Designed CNN Architecture

**Figure 3:**
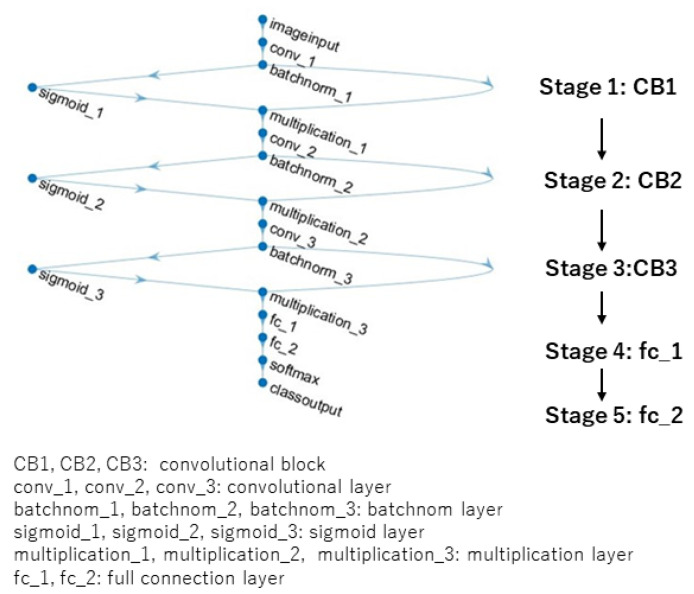
A new designed architecture.

Inspired by the attention mechanism and the architecture of EffcientNetB0 [37], a new CNN architecture is designed for the image AS classification. The architecture is shown in Figure 3.

This architecture consists of three convolutional blocks, CB1, CB2, and CB3, and two full connection layers, fc_1 and fc_2. The elements of the convolutional block (CB) include a convolutional layer, a batchnom layer that executes the batch normalization, a sigmoid layer, and a multiplication layer that executes the element-wise multiplication of the elements in the batchnom layer and the sigmoid layer. By adjusting the parameters of this architecture, four kinds of networks are constructed, which are all called model B1, B2, B3, and B4. In particular, for model B3 and model B4, the resolution of the input images is set up to 192 × 192. This is for the purpose of setting the resolution of the input to the fc_1 as 6 × 6, which can embody the composition regarding the rule of thirds. The details of these networks are shown in Table 1, Table 2, Table 3 and Table 4. The conv1 × 1 means that the size of the filter of the convolutional layer is 1 × 1, and the conv3 × 3 means the size of the filter is 3 × 3. Moreover, the number of the filters are 128 for CB1, 96 for CB2, and 96 for CB3. Accordingly, the channels of stage1, stage 2, and stage 3 are 128, 96 and 96, respectively. The number of the nodes of the two full connection layers are 36 and 8, respectively. Accordingly, the channels of stage 4 and stage 5 are 36 and 8, respectively. On the other hand, the stride of filter for CB1 is 8, and the stride for CB2 is 4. Accordingly, the resolution of the input to CB2 and CB3 is 28 × 28 and 7 × 7 for model B1 and model B2, respectively; the resolution of the input to CB2 and CB3 is 24 × 24 and 6 × 6 for model B3 and model B4, respectively.

### 3.3. Ensemble

On the basis of the photography principles, the ensemble of model A and model B is executed to improve the performance of the AS prediction. In detail, the probability of assigning an image to an AS class by a classification model is denoted as $p_{s}^{model}$, where *model* indicates the model type, and *s* represents the AS class. Therefore, the ensemble of model A and model B is calculated by the equation below:

(1) $$p_{s}^{ensemble}=w_{1}p_{s}^{model\;A}+w_{2}p_{s}^{model\;B}$$

where $w_{1}\;and\;w_{2}$ are the weights of $P_{s}^{model\;A}$ and $P_{s}^{model\;B}$, respectively. Then, the predicted score after the ensemble is obtained by the following expression:

(2) $$\mathrm{score}=\underset{s}{\mathrm{argmax}\{}p_{s}^{ensemble},\;s\in \left[2,\;9\right]\}$$

### 3.4. FFP and AIR

**Figure 4:**
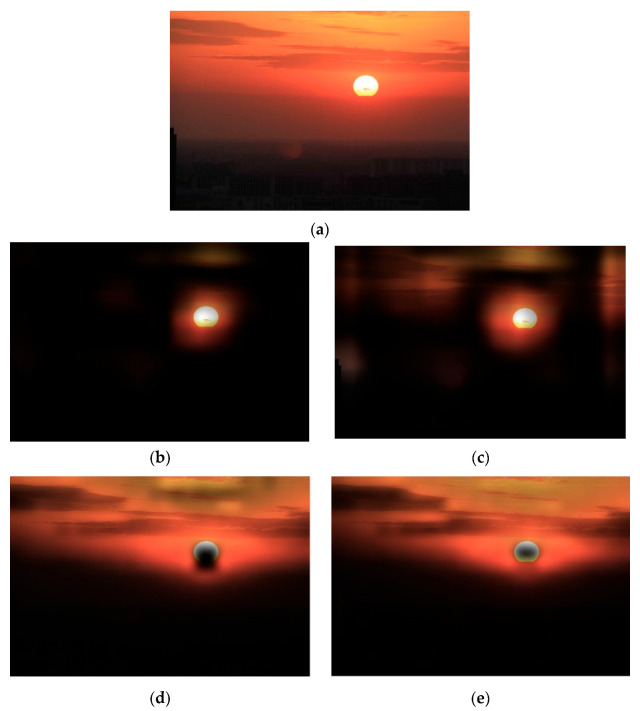
Examples of FFP and AIR. (**a**) Original image. (**b**) FFP by model A. (**c**) AIR by model A. (**d**) FFP by model B. (**e**) AIR by model B.

The FFP and AIR of model A or model B are calculated by Dai [20]. An example of the image obtained by model A and model B, regarding its FFP and AIR, is shown in Figure 4.

Model A is trained by transfer learning based on the resNet18, and model B is trained based on the architecture of model B3. The score assigned by model A is 5, and the score assigned by model B is 4. Meanwhile, the ground truth of this image in the XiheAA dataset is 4. From the results, it can be seen that the FFP and the AIR extracted by model A is the sun, and that this is the subject of the image rather than its surroundings; meanwhile, the FFP and AIR extracted by model B is the red cloud around the sun that reflects the composition of the image. The sun is in a position that seems to meet the rule of thirds, so that the score assigned by model A is higher than the ground truth. Meanwhile, the layout of the cloud is dull, so that the score assigned by model B is 4, which is the same as the ground truth. This observation is consistent with the above expectation that model A extracts the subject of the image for predicting an image’s AS, and model B extracts the holistic composition for the prediction.

## 4. Experiments and Analysis

### 4.1. Implementation Details

The proposed method is implemented in a MATLAB environment, using the MATLAB language. The image processing toolbox, the deep learning toolbox, and the machine learning toolbox are utilized for training the CNN-based models. The computer configuration is Alienware x17 R2, including 12th Gen Intel^®^ Core™ i7-12700H CPU, 32GB RAM, and NVIDIA^®^ GeForce RTX™ 3060 8GB GPU. 

### 4.2. Experimental Results of Single Models

Metrices of precision, recall, F1, and accuracy are used to evaluate the performance of the models.

**Figure 5:**
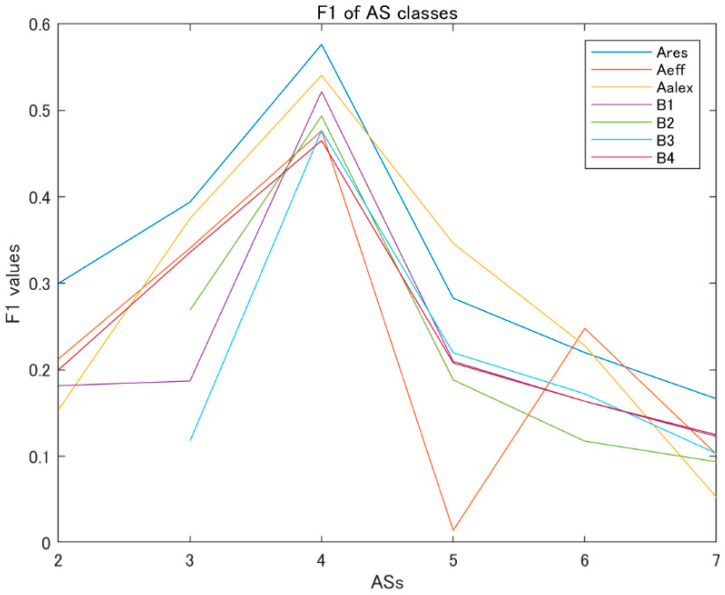
F1 values for various single models.

The F1 values of the AS classes for the various single models on the test dataset of XiheAA are shown in Figure 5. Ares, Aeff, and Aalex indicate the model A type, trained by the transfer learning based on the pretrained models resNet18, efficientNetB0 and alexNet, respectively. B1, B2, B3, and B4 indicate the model B type, with the architectures of model B1, model B2, model B3 and model B4, respectively. The assigned scores are in the range of [2,7].

For the model A group, the model Ares outperforms the other models. For the model B group, models B1, B3 and B4 outperform model B2 for predicting the highly rated images. However, model B4 outperforms the others for predicting the lowly rated images, especially model B2 and B3. From the above observation, it seems that model B4, with its architecture possessing an input size of 192 × 192, and a last convolutional layer filter size of 3 × 3, is most suitable for the AS classification. However, for the model A group, the model Ares is best for the AS classification, trained by transfer learning that is based on the resNet18. 

**Figure 6:**
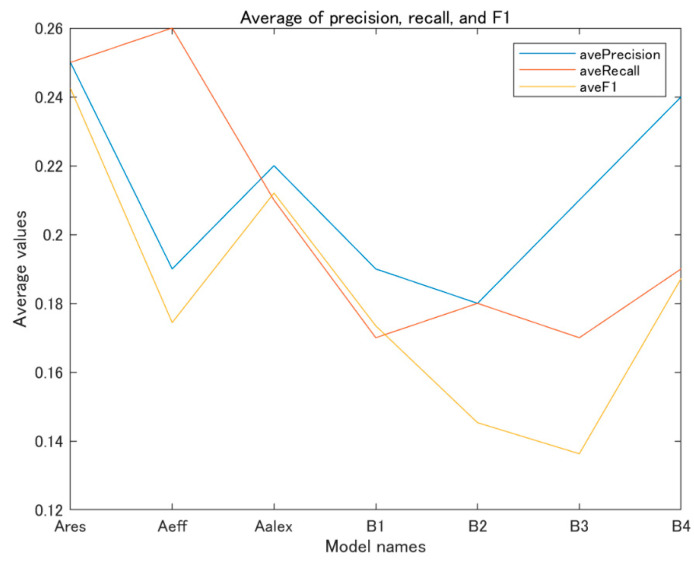
Average of precision, recall, and F1.

Figure 6 shows the average values of precision, recall, and F1 of the various models for all the AS classes on the test dataset of XiheAA.

For the precision, the average values of the model Ares and model B4 are comparatively high. For the recall, those of the model Ares, model Aeff and the model B4 are comparatively high. For the F1, which reflects the comprehensive performance of the classification, the average values of the model Ares, model Aalex and model B4 are higher. Accordingly, it can be seen that the model Ares in the A group and model B4 in the B group have the best performance for the AS classification.

### 4.3. Ensemble

**Figure 7:**
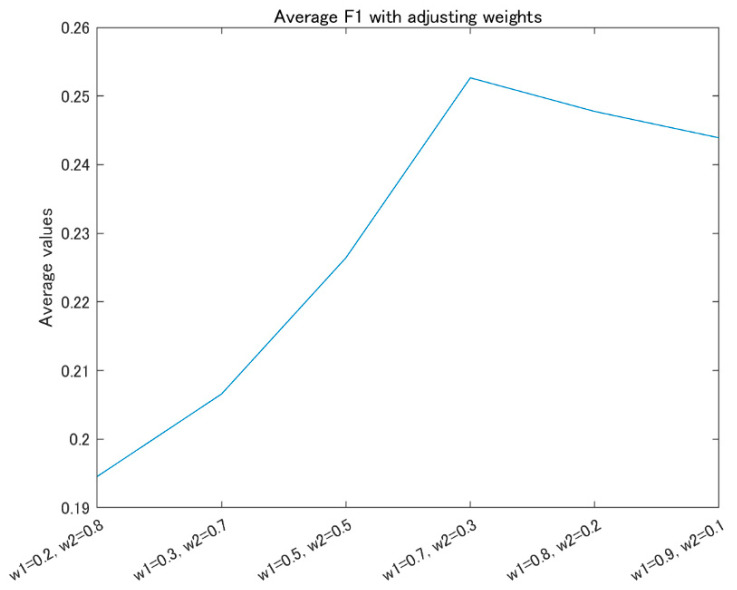
Average F1 with adjusting the weights.

Based on the above observation, the model Ares and model B4 are used for the ensemble, based on Equation (1). Then, the AS of the image is predicted by the Equation (2). Figure 7 shows the average F1 values of the AS classes on the test dataset of XiheAA, with adjusting the weights $w_{1}$ and $w_{2}$.

It is obvious that the average F1 is maximal when $w_{1}=0.7$ and $w_{2}=0.3$. The value of this is 0.253. However, the average value of F1 of the model Ares is 0.24, and that of model B4 is 0.19. Accordingly, the average F1 of the ensemble improves 5.4% compared to the model Ares, and 33.1% compared to model B4. Moreover, it can be seen that the weight of the Ares is larger than that of model B4, while the average F1 is maximal. That is, the influence of model A is stronger than model B in the AS prediction, although the ensemble can improve the above performance.

### 4.4. Experimental Results on CUHK-PQ Dataset *[1]*

The CUHK-PQ dataset [1] is used for the out-of-distribution validation. The CUHK-PQ dataset contains 10,524 high-quality images and 19,166 low-quality images. Therefore, the images predicted to have a score lower than five are assigned to the low class, while the others are assigned to the high class. Table 5 shows the overall accuracy and the averages of precision, recall, and F1 for the various models and ensembles.

From Table 5, it can be seen that the performances of the models Ares and Aeff outperform the model Aalex for the model A group. For the model B group, the performances of the models B1, B3, and B4 are almost same, and outperform model B2. For the ensemble, 0.7 Ares + 0.3 B1 indicates that the model Ares and the model B1 are used, and the weights of them are 0.7 and 0.3, respectively. Here, 0.7 Ares + 0.3 B3 and 0.7 Ares + 0.3 B4 are the analogized ones. From the results of the ensembles, it can be seen that the performances of the above ensembles are almost same. The accuracies are higher than all the single models. The improved rates are in the range of [3.6%, 8.2%]. However, the average precisions are slightly lower than the model Aeff, and the average recalls and the average F1s are slightly lower than the models Ares and Aeff, although they are obviously higher than the model B groups.

As the whole, constructing model Ares and model B4, and then taking the ensemble of these two models, is expected to improve the accuracy of the AS prediction; however, it seems not to be necessary to take the ensemble of these models in the view of the F1.

On the other hand, for the state-of-the-art research, there are some algorithms trained on the CUHK-PQ dataset. According to the description of Ref. [1], the overall accuracies of the prediction are in the range of 88.79% to 92.59%, which are about 25% higher than the results of Table 5. However, it should be pointed out that the models listed in Ref. [1] are trained and tested on the same CUHK-PQ dataset. However, the models in this paper are trained on the XiheAA dataset, and tested on the CUHK-PQ dataset. Moreover, the criteria with which the images are assigned to either the high-quality category or the low-quality category are not clear for the CUHK-PQ dataset. It is observed that some images in the high-quality category seem to not meet the photography principles, and vice versa. Therefore, it does not seem to make much sense to compare the proposed method with the state-of-the-art algorithms purely based on the accuracy of the CUHK-PQ dataset. 

### 4.5. Visualization of FFP and AIR

**Figure 8:**
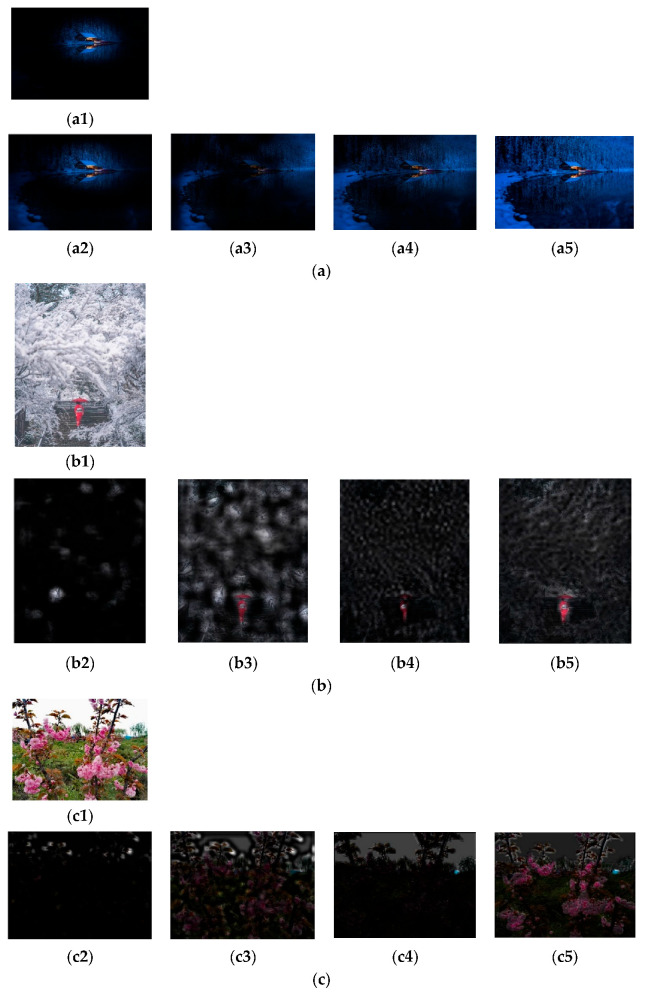
Several examples of the images’ FFPs and AIRs. (**a**) (**a1**) Original image. (**a2**) FFP by Ares. (**a3**) AIR by Ares. (**a4**) FFP by B4. (**a5**) AIR by B4. (**b**) (**b1**) Original image. (**b2**) FFP by Ares. (**b3**) AIR by Ares. (**b4**) FFP by B4. (**b5**) AIR by B4. (**c**) (**c1**) Original image. (**c2**) FFP by Ares. (**c3**) AIR by Ares. (**c4**) FFP by B4. (**c5**) AIR by B4.

Several visualization examples of the images’ FFPs and AIRs, extracted based on model Ares and model B4, are shown in Figure 8. The images are rated to score 7, score 4, and score 2, respectively. Moreover, images a and c are assigned to score 7 and 2, by either Ares or B4, respectively, while image b is assigned to score 3 by Ares, and to score 4 by B4.

Similar to Figure 8, by observing the results of approximately 300 images, it can be seen that the FFPs and the AIRs extracted by the Ares are the objects in the images, while those extracted by model B4 seem to reflect the latent layouts of the objects. Moreover, it seems that the images are assigned to the highly rated AS classes, such as with image a, if the FFPs and the AIRs meet the photography composition principles; the images are assigned to the lowly rated AS classes, such as with image c, if the FFPs and the AIRs do not meet the photography composition principles; the images are assigned to the medium-rated AS classes, such as with image b, if the FFPs and the AIRs appear to have mediocre layouts. Whether or not the correct predictions are made, the above observations are similar. It is indicated that the models trained on XiheAA seem to learn the latent photography principles, but it cannot be said that they learn the aesthetic sense.

## 5. Conclusions

In this paper, on the basis of the photography principles, we proposed a framework that constructs two types of IAA models with different CNN architectures and improves the performance of image AS prediction by the ensemble; the framework also analyzed the effectiveness of the proposed methods on the XiheAA dataset and the CUHK-PQ public dataset. Moreover, it was found that the AS classification models trained on the XiheAA dataset seem to learn the latent photography composition principles by analyzing the FFPs and AIRs of the models in the images; however, it cannot be said that they learn the aesthetic sense. On the other hand, although the precision, the recall, and the F1 of the AS prediction cannot be said to be satisfied, it is sure that the proposed framework for the AS prediction is effective. The performance of the AS prediction could be improved if more samples with higher rates or lower rates are collected to train the AS classification models.

This study has broad application prospects. The success of the image aesthetic assessment will promote the development and use of application software, such as on-line photography training, the assessment and sale of personal photography works, and a redesign from image to product.

## Figures and Tables

**Table 1:** Model B1.

Stage	Operator	Resolution	Channels
1	CB1, conv1 × 1	227 × 227	128
2	CB2, conv1 × 1	28 × 28	96
3	CB3, conv1 × 1	7 × 7	96
4	fc_1	7 × 7	36
5	fc_2	1 × 36	8

**Table 2:** Model B2.

Stage	Operator	Resolution	Channels
1	CB1, conv1 × 1	227 × 227	128
2	CB2, conv1 × 1	28 × 28	96
3	CB3, conv3 × 3	7 × 7	96
4	fc_1	7 × 7	36
5	fc_2	1 × 36	8

**Table 3:** Model B3.

Stage	Operator	Resolution	Channels
1	CB1, conv1 × 1	192 × 192	128
2	CB2, conv1 × 1	24 × 24	96
3	CB3, conv1 × 1	6 × 6	96
4	fc_1	6 × 6	36
5	fc_2	1 × 36	8

**Table 4:** Model B4.

Stage	Operator	Resolution	Channels
1	CB1, conv1 × 1	192 × 192	128
2	CB2, conv1 × 1	24 × 24	96
3	CB3, conv3 × 3	6 × 6	96
4	fc_1	6 × 6	36
5	fc_2	1 × 36	8

**Table 5:** The overall accuracy and the averages of precision, recall, and F1.

Model	Accuracy	avePrecision	aveRecall	aveF1
**Ares**	0.650	0.628	0.636	0.630
**Aeff**	0.633	0.641	0.654	0.628
**Aalex**	0.619	0.578	0.576	0.576
**B1**	0.615	0.576	0.575	0.575
**B2**	0.577	0.546	0.548	0.546
**B3**	0.617	0.576	0.573	0.573
**B4**	0.618	0.576	0.572	0.573
**0.7 Ares + 0.3 B1**	0.673	0.634	0.617	0.621
**0.7 Ares + 0.3 B3**	0.674	0.636	0.617	0.621
**0.7 Ares + 0.3 B4**	0.674	0.636	0.617	0.621

## Data Availability

Not applicable.

## Abbreviations

CNN	Convolution Neural Network
RSRL	Repetitively Self-Revised Learning
IAA	Image Aesthetics Assessment
AS	Aesthetic Score
FFP	First Fixation Perspective
AIR	Assessment Interest Region
GT	Ground Truth
CB	Convolutional Block
